# Fly Ash, from Recycling to Potential Raw Material for Mesoporous Silica Synthesis

**DOI:** 10.3390/nano10030474

**Published:** 2020-03-05

**Authors:** Marius Gheorghe Miricioiu, Violeta-Carolina Niculescu

**Affiliations:** National Research and Development Institute for Cryogenics and Isotopic Technologies-ICSI Ramnicu Valcea, 4th Uzinei Street, 240050 Ramnicu Valcea, Romania; marius.miricioiu@icsi.ro

**Keywords:** coal combustion, fly ash, mesoporous silica, recycling

## Abstract

In order to meet the increasing energy demand and to decrease the dependency on coal, environmentally friendly methods for fly ash utilization are required. In this respect, the priority is to identify the fly ash properties and to consider its potential as raw material in the obtaining of high-value materials. The physico-chemical and structural characteristics of the fly ash coming from various worldwide power plants are briefly presented. The fly ash was sampled from power plants where the combustion of lignite and hard coal in pulverized-fuel boilers (PC) and circulating fluidized bed (CFB) boilers was applied. The fly ash has high silica content. Due to this, the fly ash can be considered a potential raw material for the synthesis of nanoporous materials, such as zeolites or mesoporous silica. The samples with the highest content of SiO_2_ can be used to obtain mesoporous silica materials, such as MCM-41 or SBA-15. The resulting mesoporous silica can be used for removing/capture of CO_2_ from emissions or for wastewater treatment. The synthesis of various porous materials using wastes would allow a high level of recycling for a sustainable society with low environmental impact.

## 1. Introduction

**Figure 1:**
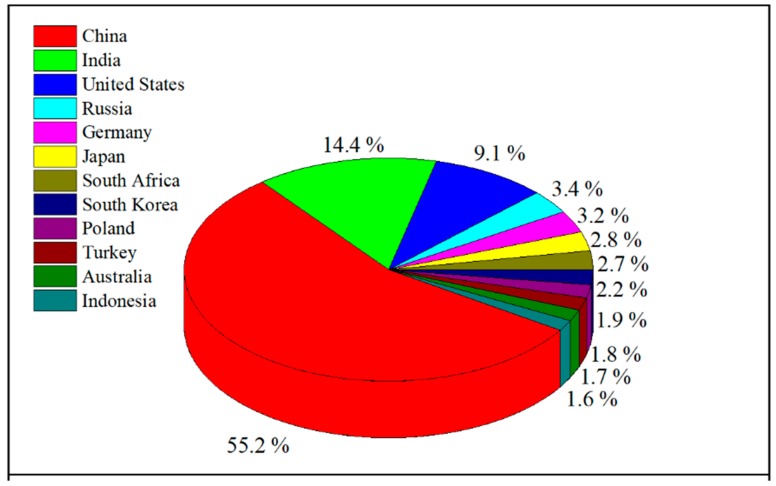
Global coal consumption (Mt) in 2018.

Global coal consumption rose with 0.9% in 2018, the main contributors being India and China, followed by Turkey and Russia [1] (Figure 1). Due to power plants and some industrial sectors (such as steel, chemicals and cement) China is responsible for approximately half of global coal consumption [1], this value reaching about 3770 Mt, which represented 55% of the worldwide consumption in 2018, approximately four times higher than in 1990 [1].

**Figure 2:**
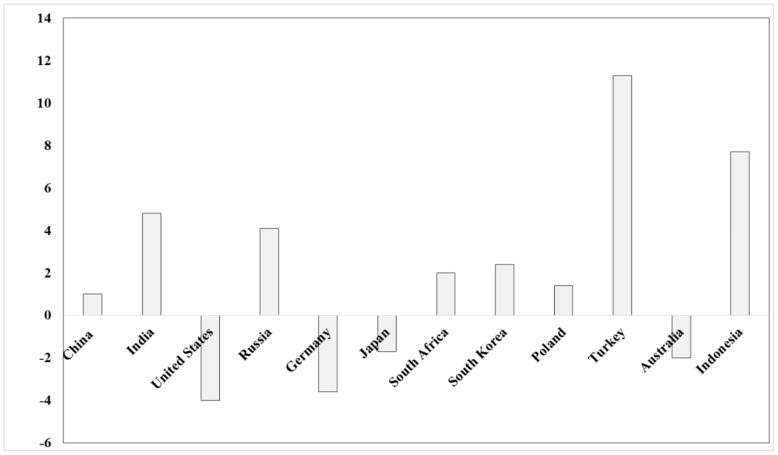
Coal consumption trend (%) in 2017–2018.

By contrast, the United States reached the lowest level in the last 40 years, the coal consumption decreasing 4% in 2018 due to the availability of natural gas at lower prices and to stronger emission legislation (Figure 2).

CO_2_ emissions are strongly related to coal consumption, and consequently the United States was expected to reduce CO_2_ emissions by 2.2% in 2019 by further reducing coal consumption, and by 3.6% in 2020 [2].

**Figure 3:**
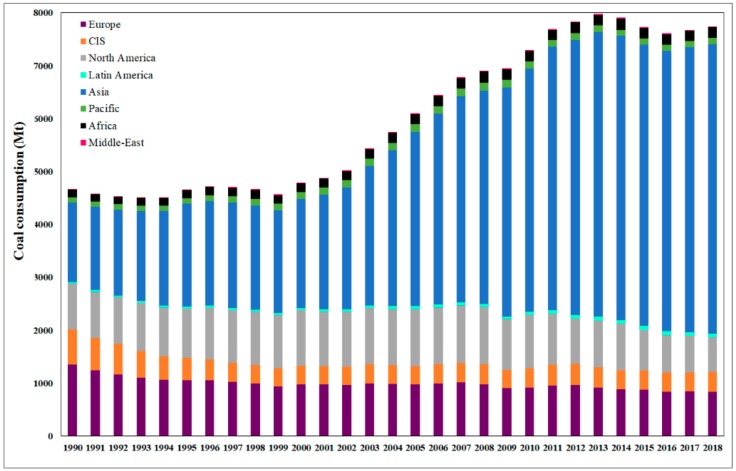
Global coal consumption trend (Mt) in 1990–2018.

Climate policies, renewable gas and CO_2_ emissions costs have been responsible for a decrease of coal consumption in the last 6 years in Europe (Figure 3). Only Turkey was an exception, due to coal consumption that increased by 11% in 2018 [1].

Regarding the statistics for the last 30 years (Figure 3), it can be observe that the coal consumption in Asia slightly increased during 1990–1995, reaching approximately 2000 Mt/year, and became constant until 1999, while in Europe consumption continuously and slightly decreased, reaching approximately half of the consumption value recorded in Asia. From 2000 to 2013, coal consumption has shown a significant increase in Asia, becoming constant until 2018 (more than 3 times higher than in the 1990s), meanwhile, in Europe, it varied slightly.

Due to coal consumption decreasing, the capture and storage of CO_2_ from emissions released into the atmosphere using new and cheap materials with high properties can be considered a reliable solution for greenhouse gases reduction. The most efficient adsorbents for CO_2_ reported in literature are zeolites, porous silica and active carbons [3].

Along with the increase of CO_2_ resulting from coal consumption, a considerable amount of fly ash is obtained as by-product of coal combustion. The fly ash is widely used as raw material in the cement industry. However, if the coal fly ash exceeds the worldwide demand it could be a problem due to storage spaces. For example, in China the utilization rate increases directly with the increase of the amount of fly ash obtained, reaching 70% 2015 [4]. In India, the utilization rate registered a significant increase in the period 2001–2011, reaching 62% [4].

The same tendency was observed in the USA, where the utilization rate reached about 50% in 2015 [4]. According to the American Coal Ash Association, the percent of used fly ash increased from 40% in 2000 to 60% in 2018, this being normal due to the coal consumption decrease [5]. In Russia, the utilization of fly ash in the period 1990–2005 was approximately 19%, the main application being in the cement industry [6]. Also, the consumption trend could increase due to the attention given to geo-polymer materials production [7]. Therefore, it is necessary to find some applications for coal fly ash use.

In the last decade, fly ash was used as raw materials for obtaining zeolites X (FAU framework type) [8,9], Y (FAU) [10], A (LTA) [8,9,11] or ZSM-5 (MFI) [12]. Furthermore, the synthesis of mesoporous silica from fly ash has also attracted interest due to the resulting material characteristics [13,14,15,16,17,18]. Mesoporous silica materials obtained from fly ash are considered to surpass the limitations of the microporous zeolites in the removal of macromolecule pollutants by adsorption [13].

The mesoporous silica materials, known since 1992 as M41S, present great potential in worldwide applications, such as catalysis or wastewater treatment, due to their properties, namely, uniformity of pore distribution (with size between 2 and 50 nm), high surface area, (around 1000 m^2^/g) and good stability in thermal conditions [19,20].

A close survey of the data highlights the fact that the chemical composition of fly ash (mainly silica and alumina compounds) is significantly different. Moreover, the synthesis conditions, including the pre-treatment step, are often not completely described or some inconsistencies among the literature sources were observed.

This overview systematically explores the synthesis of various mesoporous silica materials derived from fly ash by taking into consideration the waste properties (fly ash), pre-treatment procedures or the hydrothermal treatment parameters (temperature, time and substrate concentrations).

## 2. Fly Ash Properties

Fly ash is a complex material, being a by-product resulting from the combustion of various coals with high contents of minerals [4,21,22,23]. Consequently, fly ash is rich in metallic oxides, in the order SiO_2_ > Al_2_O_3_ > Fe_2_O_3_ > CaO > MgO > K_2_O and large amount of unburned carbon. Furthermore, fly ash contains trace elements that can have a negative impact on the environment [24,25,26]. These can easily migrate from fly ash, through interaction with water, conducting to the soil and ground water contamination with heavy metals such as Cr, V, Ni, Cd and Pb [27]. Also, wind action contributes to the environmental pollution, by spreading the ash particles in the air.

**Figure 4:**
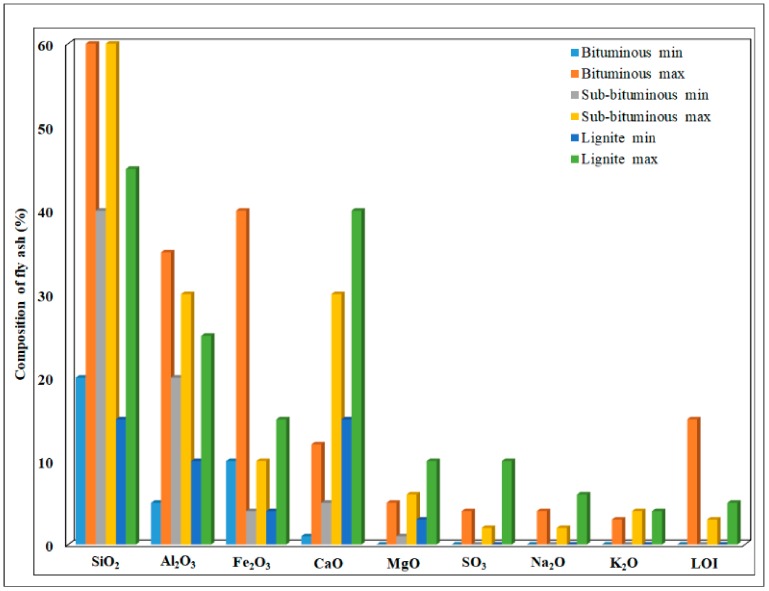
Different coal fly ash composition.

As it is shown in Figure 4, the content of metallic oxides in fly ash is depended by the coal type. Thus, the SiO_2_ contents were higher in the fly ash derived from sub-bituminous (40%–60%) and bituminous (20%–60%) samples than in lignite samples (15%–45%). The same trend was seen for Al_2_O_3_. In the case of CaO and MgO higher contents were observed in fly ash from lignite samples (15%–40% and 3%–10%, respectively), followed by sub-bituminous samples.

Small contents of SO_3_, Na_2_O and K_2_O were observed in all the fly ash samples. The highest percent value for unburned carbon, determined by its loss-on-ignition (LOI), was found in bituminous fly ash (15%).

Comparing the sample contents, it can be highlighted that all types of fly ash are rich sources of SiO_2_ and Al_2_O_3_, their recovery being an issue raised by the waste management. Also, the high content of CaO can be used for CO_2_ capture and permanent sequestration, resulting in CaCO_3_ [27]. Bituminous fly ash could be a precursor for activated carbon sorbent.

The intensive investigations carried out for fly ash reuse have resulted in the development of techniques for producing glass ceramics, ceramic wares, silicon carbide, silicon nitride, hollow/masonry/concrete blocks, cordierite or mullite. Recently, fly ash was applied in the development of mesoporous silica for CO_2_ capture [28].

## 3. Environmental Risk Assessment

The reuse of fly ash must be encouraged for many reasons. For example, the disposal costs would be minimized; also, less landscape would be reserved for its disposal; and the by-products may be used as raw materials.

Fly ash may have metal concentrations up to 10 times higher than coal [4]. As it was already mentioned, there are many natural factors (e.g., rain, wind) that contribute to interaction of the metals with humans, reaching significant concentration in soil and water and finally in crops. This process is directly dependent on several parameters, such as particle size, pH, interaction time, trace elements concentration in fly ash [4].

**Figure 5:**
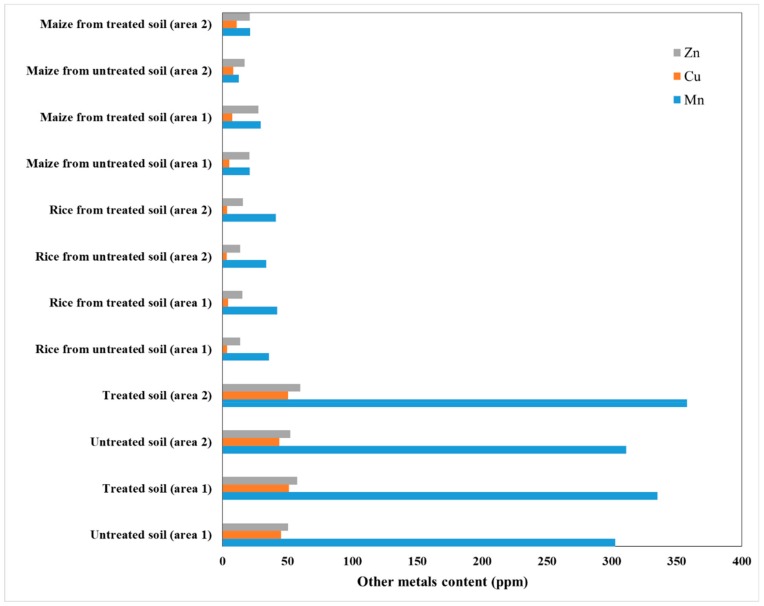
Heavy metal contents in soil, rice and maize from two areas, treated and untreated with fly ash.

**Figure 6:**
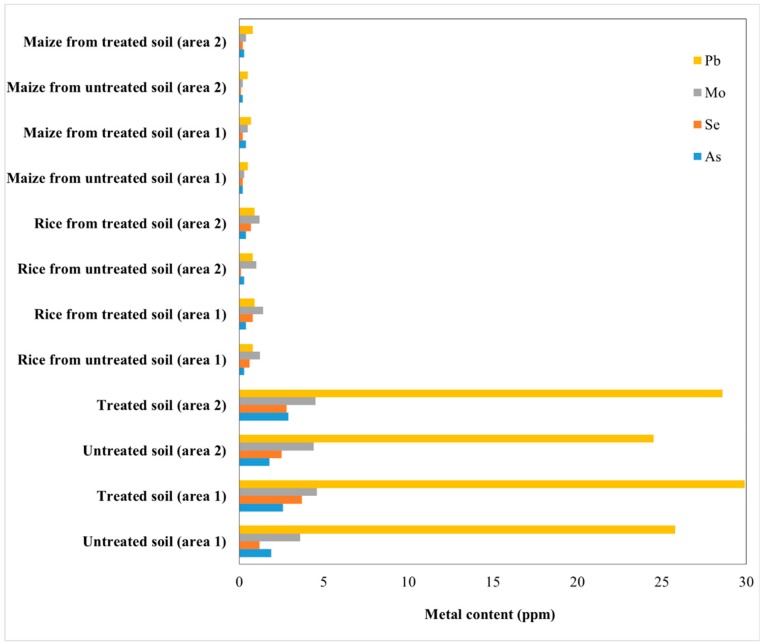
Toxic elements contents in soil, rice and maize from two areas, treated and untreated with fly ash.

**Figure 7:**
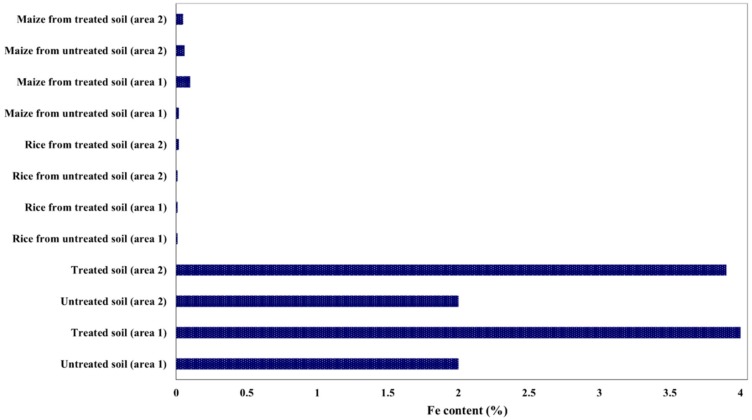
Fe content in soil, rice and maize from two areas, treated and untreated with fly ash.

A typical metallic composition in fly ash collected from different power plants in India is presented in Table 1. Furthermore, the effect of fly ash on soil quality for maize and rice crops was investigated, in two different cultivation areas from the eastern part of India and the results are presented in Figure 5, Figure 6 and Figure 7 [29,30]. The fly ash was dried and mixed with cellulose to obtain pellets and was applied to the soil (about 200 t/ha).

As it can be seen from Figure 5, the soil enrichment with heavy metals in rice crops did not exceed the upper control limits (in soil: 1500 ppm for Mn, 100 ppm for Cu and 300 ppm for Zn; in plant: 300 ppm for Mn, 50 ppm for Cu and 100 ppm for Zn) [31,32,33]. Thus, the soils treated with fly ash presented a slight enrichment with microelements, such as Zn, Mn and Cu, which are essential for plant growth. Also, the enrichment of rice and maize crops with microelements was observed. In the case of rice crops, the enrichment with Cu was lower than with Zn and Mn.

Regarding the concentration of toxic elements in soil and crops, Figure 6 shows that the Mo concentration reached almost the critical level, being a concern for the environment. The differences between the rest of the toxic element concentrations (Pb, Se, As) and their upper control limits are higher (in soil: 200 ppm for As, 10 ppm for Se, 5 ppm for Mo and 100 ppm for Pb; in plant: 30 ppm for As, 0.69 ppm for Se, 1.2 ppm for Mo and 0.87 ppm for Pb), without significant risk to the crops.

It was observed that Fe was the major element. As it can be seen in Figure 7, there are significant differences between the enrichment of Fe in crops from untreated and treated soils (upper control limit for Fe being 5.6% in soil and 0.2% in plant).

It was also demonstrated that the soil treated with fly ash contributed to the increase of crop yields, by the action of essential plant nutrients, such as Mg, S, K, Ca, Mn, Fe and Zn [29]. The fly ash can modify the physical properties of the soil, such as porosity and density, increasing the water retaining capacity [29,34]. Due to low concentration of toxic elements in fly ash, the critical levels were not reached and the grains were considered safe for consumers.

As it can be seen, the use of coal fly ash meets technical, economic and legal barriers. The main technical milestones refer to coal fly ash production, specifications and standards, product demonstration and commercialization.

For coal-using plants, the incomes from the sale of fly ash are often negligible, the most important economic barriers coming from the increased cost of transportation of fly ash and competition from locally available natural sources. Legal barriers resulting from the lack of knowledge regarding the potential ash application, insufficient data on environmental and health risks, lack of regulations and procurement guidelines.

Worried industry and government representatives, scientists, and engineers have created national and international organizations to overcome the milestones of coal fly ash reuse. An integrated approach must be designed in order to produce a superior quality of fly ash-based materials, which can satisfy the consumer’s expectations. Furthermore, high quantities of fly ash are often followed by emission of greenhouse gases, significantly intruding global warming. As a consequence, to conform to the environmental requirements, major efforts must be achieved in fly ash management, reducing the negative effect on the environment.

A deep analysis on the cost of fly ash use versus conventional building materials is required, being necessary to apply the best engineering practices in order to minimize as much as possible the environmental risk.

## 4. Fly Ash as Raw Material for Mesoporous Silica Synthesis

In order to increase the recycling of the coal fly ash and to reduce the environmental risk of contamination with heavy metals, several applications for recovery of valuable components from fly ash were developed.

Thus, among the fly ash used in conventional applications (cements obtaining), various mesoporous silica materials with improved properties were obtained, able to be applied in environmental depollution, namely CO_2_ reduction and wastewater treatment.

The methods for obtaining these materials from fly ash were basically similar, the differences consisting in varying different parameters such as: pH, the temperature and the time of hydrothermal or aging treatment and also the quantities of raw materials or reagents. These variations influenced more or less the obtained material properties.

The used coal fly ash for the silica synthesis had various particle sizes (2.0–30 µm) and chemical compositions (31.6–60.1 wt.% Si, 10.2–40.8 wt.% Al, 0.8–8.4 wt.% Fe, 0.4–24.8 wt.% Ca) [35,36,37,38].

Porous materials, such as Al-MCM-41 and SBA-15, were obtained from coal fly ash, and they were used as catalysts in the cumene cracking reaction [35]. In this respect, the fly ash was treated with an aqueous solution of NaOH under thermal condition; the supernatant obtained after the resulting suspension filtration was used as a silica source for MCM-41. Its composition, beside 11,000 ppm Si, was rich in Na and Al, with 35,000 and 380 ppm, respectively. It was demonstrated that the pH adjustment has directly influenced the Al-MCM-41 synthesis. The Al incorporation was poor in the case of SBA-15, compared with the MCM-41, remaining in the dissolved state in the supernatant due to the high acidic medium. Furthermore, the Al-MCM-41, derived from both fly ash and pure chemicals (obtained by a conventional method), was tested for a cumene cracking reaction involving a high acid medium [35]. Despite the small differences of physical properties of the two mesoporous materials (surface area: 842 and 940 m^2^/g, pore volume: 0.75 and 0.85 cm^3^/g and pore diameter: 3.7 and 2.7 nm) it was observed that the Al-MCM-41 from the conventional method was more efficient in the cumene conversion because not all of the Al presented in the fly ash-derived porous material had catalytic activity. Thus, the cumene conversion was threefold higher, ~22%, at the beginning of the reaction for the Al-MCM-41 prepared from pure reagents and decreased drastically during the first hour, slowly decreasing the next hour for the two materials, reaching 6%, two times higher than Al-MCM-41 derived coal fly ash.

MCM-41 was also obtained by adapting the mesoporous synthesis from the previous study [35], with moderate modification of the parameters, such as temperature and time [36]. Also, the catalytic performance of the MCM-41 synthesized from fly ash and from conventional reagents for Mannich reaction application were compared [36]. It was supposed from the beginning that there would be superior catalytic activity of the obtained material, due to the lower particle size and higher surface area of MCM-41 than the fly ash. The materials were tested as catalysts in the Mannich reaction of acetophenone, benzaldehyde and aniline by varying the catalyst quantity (0.1 g, 0.2 g and 0.3 g) and the solvent nature (EtOH, CH_3_CN, Toluene, Tetrahydrofuran (THF), CH_2_Cl_2_ and H_2_O). A 90% yield was obtained in 4 h for 0.2 g MCM-41 in ethanol, providing a good catalytic active area for the reaction. The yield remained constant with the increase of catalyst quantity at 0.3 g. Instead, a lower efficiency, down to 50%, was observed with the decrease of catalyst quantity, to 0.1 g, even if the reaction time has been extended to 8 h [36]. The MCM-41 can be an optimal replacement for environment-unfriendly solvents used in catalysis.

A supernatant with higher contents of Si, Al and Na (15.37, 499.00 and 48.70 ppm, respectively) was obtained starting also from the method previously described [35], the fly ash (raw material) being rich in these elements [37]. The mesoporous Al-MCM-41 adsorbent obtained for the removal of methylene blue (MB) was finally synthesized by varying the ethyl acetate amount [37]. This had a direct influence on specific surface area and pore volume of the silica material, thus the Al-MCM-41 obtained by using 10 mL of ethyl acetate shown improved properties [37]. Thus, when 10 mL of ethyl acetate was used, the surface area increased from 25 to 525 m^2^/g, the pore diameter decreased from 7.54 to 5.13 nm and the pore volume increased from 0.13 to 0.71 cm^3^/g, although not reaching the properties of the material obtained through conventional methods [38]. The Al-MCM-41 capacity to adsorb the methylene blue was influenced by the pH, contact time, temperature and concentration. Regarding the contact time parameter, a drastic increase of the MB adsorption was observed, followed by a slower increase in intervals 0–10 min and 10–120 min, respectively. After 120 min, the MB reached the equilibrium adsorption level. The MB adsorption had a continuous increasing trend with the increase of pH from 3 to 10, with a higher increment in pH interval 3–7. The highest adsorption capacity was up to 277.78 mg/g and it was reached at room temperature and pH 10.

MCM-41 was also synthesized from brown and hard coal fly ash (10 samples) using two procedures, namely pulverized-fuel (PC) and fluidized-bed boilers (CFB) [39]. The X-ray fluorescence spectroscopy (XRF) revealed different chemical composition of fly ashes, the SiO_2_ and Al_2_O_3_ ranging from 46.15 wt% to 56.52 wt% and from 18.48 wt% to 31.06 wt%, respectively. The lowest and the highest value of SiO_2_ were obtained from PC combustion. Compared to other studies [35,37], where the silica content resulted in the filtrate was directly proportional with the percentage in the coal fly ash, this research highlighted a silica concentration significant lower compared to its content in the coal fly ash. In consequence, this sample could not be used for further MCM-41 synthesis because the properties of the resulting material are strongly dependent of the Al/Si ratio in the filtrate.

It was observed that the impurities presented in the coal fly ash composition, such as Fe, Ca, K, S and P, were also present in the channels of the obtained mesoporous material, conducting to poor properties compared to the commercial material [39].

For comparison, the ash resulting from the rice husk combustion was tested, using similar extraction steps as in the case of fly ash [40]. Even if the silica content was 33% higher in the rice husk ash, the MCM-41 obtained from the two raw materials had the same silica content.

It was concluded that, despite the similar chemical composition, the textural properties of the rice husk and fly ash-derived materials were superior to the commercial MCM-41, presenting higher pore volume for all synthesized materials, higher specific surface area and similar morphologies.

Also, the reaction time influenced the obtained suspension containing the surfactant (hexadecyltrimethylammonium bromide-CTAB) [40]. A longer time (96 h) was required to conduct the decrease of the surface area and pore volume. After the polyethyleneimine (PEI) impregnation, the obtained MCM-41 and a MCM-41 commercial sample were compared for CO_2_ capture. The results shown higher CO_2_ uptakes (with 2 wt.%) for the synthesized materials containing 60 wt.% PEI, the materials having faster kinetics due to larger pore volume comparing to the commercial materials.

It was established that the fly ash desilication rate can reach 46.3%, by using sodium hydroxide at a mass ratio of 1:6.4, during 4 h under thermal condition (95 °C). This solution was used as silica source to obtain SBA-15 mesoporous silica. The method involved further hydrothermal treatment for two days at 110 °C and a triblock copolymer as template-poly(ethylene oxide)/poly(propylene oxide)/poly(ethylene oxide) [41].

The functionalization of SBA-15 with amino groups involved, as in case of MCM-41, aminopropyltriethoxysilane (APTES), as source of NH_2_- groups, and toluene as solvent [41,42,43]. An application of SBA-15 silica type derived from fly ash was Pb^2+^ adsorption [41] (see Table 2).

Thus, the effect of time, temperature and Pb^2+^ initial concentration on amino-SBA-15 adsorbent was studied and it was remarked that the adsorption equilibrium time was achieved after 1 h, reaching 131 mg/g (Table 2). Also, the temperature influenced the adsorption rate, increasing drastically between 20 and 30 °C (98.40% removal efficiency) becoming constant up to 40 °C. The increase of the Pb^2+^ concentration led to a higher removal efficiency, up to 93% for a concentration of 100 mg/g.

It was demonstrated that the aluminosilicate with mesoporous structure has great potential for the removal of organic dyes (such as methylene blue or crystal violet) from wastewater. In this respect, mesoporous aluminosilicate was synthesized from fly ash by varying the Si/Al molar ratio [44]. X-ray diffraction analysis of the obtained materials revealed a well-ordered hexagonal structure, as in the case of MCM-41, direct proportionally with the increase of Si/Al ratio. Thus, the surface area (~1080 m^2^/g) and the pore volume (~0.96 cm^3^/g) increased with the increase of Si/Al molar ratio, up to 25. The efficiency of the aluminosilicate on the methylene blue and crystal violet removal from wastewater was evaluated, revealing its high adsorption capacity (Table 2). Higher adsorption capacity was obtained for mesoporous materials with a Si/Al ratio equal to 10, due to the ordered mesoporous structure and pore size distribution with narrow pores. Also, as it was already mentioned [37], the pH has a direct influence on the adsorption capacity of the mesoporous materials, the highest value being obtained for the material resulted from a solution with a pH value of 11 (1100 mg/g for methylene blue and 1400 mg/g for crystal violet) [44].

## 5. Economic Assessment

Commercial MCM-41 production is expensive, due to intense energy consumption or utilization of inorganic and organic silicate reagents, the identification of new cheap silica sources being a viable future solution [40,45]. Beside the coal fly ash, these sources can be found in various wastes, such as agriculture slag [46], electronic components [47] or rice and wheat husk [48,49].

The synthesis of silica from waste is not free of cost, because the procedure is similar to the conventional methods, involving hydrothermal treatment with intensive energy consumption. The difference between conventional and unconventional synthesis is the source of silica, which leads to extra synthesis steps. The significant reduction of the synthesis time, from 72 h (time used in conventional methods) to 2 h, could result in the cost decrease, by saving energy [50]. The estimated cost for the synthesis of MCM-41 from fly ash was about 1200 euro/kg, representing half the price of a similar commercial material [40,51]. The higher purity of this type of material could reach even a price of 5000 euro/kg [51].

Taking into account the depollution and storage costs for fly ash or other silica source wastes, it could lead to a fair price and a sustainable process.

## 6. Conclusions

In the last few decades, worldwide coal consumption has been monitored. High quantities of fly ash are discharged as a by-product from the coal power plants. The influence of fly ash on soil-crops chain (maize and rice) in different areas was demonstrated. In this respect, an effective process to recycle this waste is mandatory.

Fly ash proved to be suitable to use in environmental applications, for example by replacing activated carbon and zeolites as adsorbents for air pollutants or wastewater treatment. Its adsorption capacity mainly depends on the origin and the activation method. To date, no industrial set-up has been developed. Economic milestones must be surpassed. Taking into consideration the amorphous alumina-silicate nature of fly ash, it can be applied as raw material in various industrial reactions, such as obtaining ultramarine blue. Intensive harvesting depletes trace elements in the soil. Despite the fact that many studies were achieved for the use of fly ash as soil amendment, full-scale application has not been accomplished. In the near future, farmers may consider fly ash to be a substitute for lime to enrich the soil.

Fly ash has high silica content, which makes it a potential source for obtaining nanoporous materials, such as mesoporous silica. In this overview, various methods for obtaining mesoporous silica materials from fly ash were examined by considering the ash composition, pretreatment procedures, and synthesis conditions, in order to identify the optimal parameters for synthesis. The physical properties of the materials derived from fly ash confirmed their mesoporous nature, similar to the mesoporous silica materials obtained by conventional methods. The mesoporous silica materials with a high surface area and large pore volume derived from fly ash can be applied as a support or as a surface-functionalized host for capturing CO_2_ from gaseous emissions or for various water pollutants. The synthesis of different nanoporous materials using as raw materials various wastes would enable a high level of recycling for a sustainable society with a low environmental impact.

## Figures and Tables

**Table 1:** The elemental composition of the fly ash.

Element	Fe	Mn	Cu	Zn	As	Se	Mo	Pb
Unit	%	ppm	ppm	ppm	ppm	ppm	ppm	ppm
Concentration Range	2–3	148–261	64–83	103–150	3–6	2–3	3–4	15–40

**Table 2:** Applications of the synthesized mesoporous silica.

No.	Mesoporous Silica	Application	Reaction Conditions	Efficiency/Yield	Ref.
1	Al-MCM-41	Catalyst-cumene cracking reaction	T = 623 K	~ 3%–6 %	[35]
			W/F = 0.2 h (contact time)		
			carrier gas flow (N_2_) = 40 mL/min		
			cumene partial pressure = 7.9 kPa		
2	MCM-41	Catalyst—Mannich reaction	acetophenone = 5 mmol	90 %	[36]
			benzaldehyde = 5 mmol		
			aniline = 5 mmol		
			MCM-41 = 0.2 g		
			ethanol = 20 mL		
			t = 4 h (reaction time)		
3	Al-MCM-41-10	MB (methylene blue) adsorbent	T = 298 K	278 mg/g	[37]
			T = 308 K	276 mg/g	
			T = 318 K	272 mg/g	
			pH = 3 (at 293 K)	~137 mg/g	
			pH = 5 (at 293 K)	~156 mg/g	
			pH = 7 (at 293 K)	~175 mg/g	
			pH = 10 (at 293 K)	~181 mg/g	
			t = 2 min (adsorption time)	~223 mg/g	
			t = 10 min (adsorption time)	~255 mg/g	
			t = 120 min (adsorption time)	~266 mg/g	
			t = 480 min (adsorption time)	~267 mg/g	
4	MCM-41 Commercial + 50% PEI	CO_2_ adsorption	T = 348 Kgas concentration = 15 % CO_2_	8.37 wt.% CO_2_ uptake	[40]
	MCM-41 Commercial + 60% PEI			11.17 wt.% CO_2_ uptake	
	MCM-41 PFA-1 + 50 % PEI loading			10.64 wt.% CO_2_ uptake	
	MCM-41 PFA-1 + 60 % PEI loading			13.08 wt.% CO_2_ uptake	
	MCM-41 PFA-2 + 50 % PEI loading			8.79 wt.% CO_2_ uptake	
	MCM-41 PFA-2 + 60 % PEI loading			12.91 wt.% CO_2_ uptake	
	MCM-41 RHA + 50 % PEI loading			10.13 wt.% CO_2_ uptake	
	MCM-41 RHA + 60 % PEI loading			13.31 wt.% CO_2_ uptake	
5	NH_2_-SBA-15	Pb^2+^ adsorption	30 min (adsorption time)	~100 mg/g (adsorption)	[41]
			60 min (adsorption time)	~131.00 mg/g (adsorption)	
			90 min (adsorption time)	~130.00 mg/g (adsorption)	
			120 min (adsorption time)	~131.00 mg/g (adsorption)	
			180 min (adsorption time)	~131.00 mg/g (adsorption)	
			20 °C (adsorption time)	~96.51% (removal efficiency)	
			30 °C (adsorption time)	~98.40% (removal efficiency)	
			40 °C (adsorption time)	~98.50% (removal efficiency)	
			Pb^2+^ concentration = 20 mg/g	~87.5% (removal efficiency)	
			Pb^2+^ concentration = 60 mg/g	~91.5% (removal efficiency)	
			Pb^2+^ concentration = 100 mg/g	~93% (removal efficiency)	
6	Aluminosilicate (SA)	Methylene blue (MB) adsorption	475 mg/L (initial concentration)	2003 mg/g	[44]
			adsorbent loading = 0.2 mg/L		
		Crystal violet (CV) adsorption	200 mg/L (initial concentration)	458 mg/g	
			adsorbent loading = 0.2 mg/L		
